# Large animal models in the study of gynecological diseases

**DOI:** 10.3389/fcell.2023.1110551

**Published:** 2023-01-23

**Authors:** Minghua Cui, Yuehui Liu, Xiaoping Men, Tao Li, Da Liu, Yongzhi Deng

**Affiliations:** ^1^ Gynecology Department, Affiliated Hospital of Changchun University of Chinese Medicine, Changchun, Jilin, China; ^2^ Laboratory Department, Affiliated Hospital of Changchun University of Chinese Medicine, Changchun, Jilin, China; ^3^ Department of Acupuncture and Massage, The Third Affiliated Hospital of Changchun University of Chinese Medicine, Changchun, Jilin, China; ^4^ School of Pharmacy, Changchun University of Chinese Medicine, Changchun, Jilin, China; ^5^ The Third Affiliated Hospital of Changchun University of Chinese Medicine, Changchun, Jilin, China

**Keywords:** gynecological diseases, large animal models, gestational diabetes mellitus, polycystic ovary syndrome, endometriosis

## Abstract

Gynecological diseases are a series of diseases caused by abnormalities in the female reproductive organs or breast, which endanger women’s fertility and even their lives. Therefore, it is important to investigate the mechanism of occurrence and treatment of gynecological diseases. Animal models are the main objects for people to study the development of diseases and explore treatment options. Large animals, compared to small rodents, have reproductive organs with structural and physiological characteristics closer to those of humans, and are also better suited for long-term serial examinations for gynecological disease studies. This review gives examples of large animal models in gynecological diseases and provides a reference for the selection of animal models for gynecological diseases.

## Introduction

Animal models are commonly used in biomedical research as the basis for experimental and clinical hypotheses. The use of animal models is an extremely important experimental method and tool in modern biomedical research, contributing to a more convenient and effective understanding of the developmental patterns of human diseases (Robinson et al., 2019; Lunney et al., 2021). People first used animals as physiological models in ancient Greek times (Ericsson et al., 2013). Since then, animals have been utilized more and more in the study of human diseases and have gradually become an irreplaceable part. Rodents such as mice and rats reproduce rapidly and one can make them present multiple phenotypes by knockout techniques (Filipiak and Saunders, 2006). In the early 20th century, the use of rodents as models for biological research was well established. However, these rodents do not mimic all diseases because some of the physiological characteristics differ significantly from those of humans. In pursuit of higher similarity of physiological structures, large animal models such as pigs (Lunney et al., 2021), sheep (Murray et al., 2019), and horses (Schüttler et al., 2020) and other higher classes of animals have become the choice of models for disease studies.

Gynecological diseases are diseases caused by abnormal development, infections, and tumors of the female reproductive organs and mammary glands. According to the site of the disease, gynecological diseases are mainly divided into: 1) Diseases of the reproductive system such as uterine diseases and vaginal diseases, which are mostly caused by infections, but also some diseases caused by genetic factors and hormonal disorders (Grimbizis et al., 2001; Stewart, 2001; White et al., 2011); 2) breast diseases, which mostly refer to breast inflammation and malignant tumors (Harbeck and Gnant, 2017; Scott, 2022); 3) endocrine disorders due to reproductive abnormalities, such as gestational diabetes (Khalil et al., 2018). In the current research on gynecological diseases, a variety of animals have successfully simulated human diseases, and the research is divided into two main areas. The first is the mechanism of disease onset. Researchers explore the possible causes of disease through the expression of proteins associated with specific signaling pathways. In the study of signaling pathways (Lv et al., 2022), researchers can predict the potential symptoms of a disease and reveal the harm of it to other tissues or organs. The second is the treatment plan for the disease (Tyrrell, 1999). By applying spontaneous disease models or artificially induced disease models, drug efficacy is evaluated, and issues such as drug safety are assessed, which is the basis of preclinical drug research. However, due to the specificity of gynecological diseases, small rodents do not mimic the manifestations of human gynecological diseases very well (Adams et al., 2012). Large animals are often applied in the study of gynecological diseases because of their reproductive characteristics close to those of humans. In this review, we briefly introduce the application of large animal models in gynecologic diseases, using three gynecologic diseases as examples. An in-depth understanding of the application of large animal models will provide more insight into the study of gynecological diseases and enlighten researchers to pay more attention to the selection of animal models in their studies.

## Large animal models

For a long time, it has been found that it is difficult to advance medicine by using human subjects as subjects themselves. Not only are the accumulated clinical experiences limited in time and space, but many experiments are morally and methodologically restricted. The attractiveness of animal models lies in the fact that they overcome these shortcomings, and their unique role in biomedical research is being increasingly appreciated by researchers (Banstola and Reynolds, 2022). In order to fully simulate the main clinical manifestations and pathological features of a specific disease, the selection of animal models varies from disease to disease. Although rodents such as mice and rats are widely used in disease research, they have shown many shortcomings in gynecological diseases. Rodents usually have a short ovarian cycle and a luteal phase that only arises during mating, and their gestation period is much smaller than that of humans (Table 1). Their small size is also a problem, and examination of organs by necropsy does not allow for continuous follow-up of disease progression. Although there are instruments for examination of mice, such as Doppler ultrasound, the same instruments used for human examination can be applied to large animals and can be examined by palpation, which are not possible in small rodents. In contrast, gynecological diseases usually develop continuously over a specific period of time, so examination by means of dissection does not contribute to the understanding of the disease. The larger size of large animals facilitates real-time examination of the ovaries and uterus. Researchers used cows as an animal model to study the effects of aging on female reproduction. Changes in plasma estradiol concentrations and luteal phase progesterone concentrations were found to be similar to those in menopausal women (Malhi et al., 2005). Ovary-related endocrine changes in sheep have also been shown to be similar to those in women (de Souza et al., 1998). The results of large animals as models for human gynecological studies are more closely related to those of humans. Smaller animals tend to have shorter lifespans, and obtaining sufficient blood from these small animals for analysis is difficult and does not allow for long-term observation of hormone level. In addition, the pharmacokinetics of small animals differ significantly from those of humans, and the evaluation of drugs in small animals may deviate significantly from reality (Lin, 1995). In a review by Mondal et al. (2022) detailing the comparison of tumor sink between mice and pigs, it was found that using a mouse model would severely underestimate the toxicity of the drug, compared to pigs. This is due to the smaller size of the mouse, where the drug would be more preferentially concentrated at the tumor site, while the pig has higher concentrations in the plasma and greater systemic toxicity. Therefore, large animals are more advantageous in studying gynecological diseases both in terms of physiological similarity and manipulation. This is why researchers have used large animals to construct models of various gynecological diseases for basic and pharmacodynamic studies.

**TABLE 1:** Reproductive characteristics of humans and some animals. Table content was compiled from Abedal-Majed and Cupp (2019), Lu et al. (2022).

Reproductive characteristics	Women	Cows	Sheep	Mice	Monkeys
Ovarian size	4 × 3 × 1 cm	2–3 × 1–2.5 × 1–1.5 cm	1–1.5 × 0.5–1 × 0.5–1 cm	0.2 × 0.1 × 0.05 cm	1.0–1.8 × 0.4–0.6 × 0.2–0.4 cm
Diameter of ovulatory follicle	18–20 mm	15–20 mm	5–7 mm	0.9–1.1 mm	6–9 mm
Lifespan	70–80 years	10–20 years	5–15 years	1–3 years	20–25 years
Ovulatory cycle	24–30 days	17–24 days	13–19 days	4–6 days	22–33 days
Length of follicular phase	12–14 days	2–3 days	2–3 days	1–3 days	17–19 days
Length of luteal phase	14–16 days	15–18 days	12–14 days	—	13–15 days
Duration of gestation	278–282 days	278–282 days	142–148 days	21 days	156–180 days

## Gestational diabetes

Gestational diabetes (GDM) is a spontaneous hyperglycemia during pregnancy. Gestational diabetes is one of the factors that affect the prognosis of pregnancy, increasing the risk of pregnancy and causing adverse pregnancy outcomes (Bellamy et al., 2009; American Diabetes Association, 2013). In addition, GDM may have an impact on maternal health long after delivery, increasing the risk of type 2 diabetes (Peters et al., 1996) as well as cardiovascular disease (Shostrom et al., 2017; Plows et al., 2018). Therefore, an in-depth study of the pathogenesis of gestational diabetes mellitus and the proposal of targeted therapeutic approaches are of great clinical value. Establishing effective animal models is the basis for understanding GDM. In GDM modeling, chemical induction of toxicity in pancreatic β-cells of rodents is commonly used. However, rodents have a shorter gestation period, and gestational hormones and islet structure are different from humans (Schüttler et al., 2020). This is the main problem of using them for GDM studies (Pasek and Gannon, 2013; Gao et al., 2021). Pigs are similar to humans in terms of anatomy, physiology, and disease mechanisms (Yao et al., 2016). They were used to create a maternal diabetes model to explore the effects of tetraoxacillin diabetes and maternal fasting on fetal development. For the quantitative increase in body fat only in fetuses of diabetic pigs, it was concluded that diabetic pregnancy stimulates the *ab initio* synthesis of fatty acids from fetal fat and is the main mechanism for the increase in fetal body fat accumulation (Kasser et al., 1981). In Ezekwe’s study, streptozotocin was administered to pregnant sows during gestation to induce diabetes and to explore changes in fetal growth, energy reserves and body composition in neonatal pigs (Ezekwe et al., 1984). With the rapid development of genetic engineering technology, researchers are using transgenic techniques to manipulate animals and construct the desired models. Mice can be genetically edited and reproduced stably by CRISPR/Cas9 technology (Hall et al., 2018). Gene editing techniques are also used in large animals, and people have successfully used sperm-mediated gene transfer (Umeyama et al., 2012), gene-targeted technique (Watanabe et al., 2010) to get transgenic pigs, which have potential in the research of gynecological diseases. Renner et al. (2019) designed transgenic pigs with INSC93S, a genetic mutation that causes hyperglycemia and insulin resistance in pregnant pigs in late gestation, and the transgenic pigs became a promising model for GDM. In addition to pigs, dogs have also been used to model GDM. Moore et al. (1985) successfully induced a GDM in dogs by feeding them high fat and fructose at weeks six and seven of gestation. Further exploration of hepatic glucose metabolism in dogs during gestation revealed that the hepatic response to hyperglycemia is diminished in normal pregnancy whereas this response is more suppressed in the GDM model. This response may be responsible for exacerbating hyperglycemia and is one of the characteristics of GDM (Coate et al., 2013). Large animals are of greater value in the construction of gestational diabetes models (Table 2), and gene editing technology offers new directions for the construction of such models.

**TABLE 2:** Different large animals for the study of gestational diabetes.

Species	Method	Result	References
Pigs	Alloxan injection	Elevated liver glycogen concentration in offspring of gestational diabetic sows with unaffected prenatal muscle development	Ezekwe and Martin, (1978)
		Maternal diabetes induces increased abundance of IGF-I mRNA in fetal skeletal muscle, liver, heart, kidney and placenta and decreased IGF-I mRNA levels in the brain	Ramsay et al. (1994)
	Streptozotocin injection	The offspring of severely diabetic sows have elevated body lipids, while the offspring of mildly diabetic sows have unaffected body lipids	Ezekwe et al. (1984)
Dogs	High fat/fructose diet	Dogs fed a high-fat/high-fructose diet in late gestation exhibit worsened glucose tolerance and impaired systemic and hepatic insulin sensitivity	Moore et al. (1985)
Sheep	High-fat diet	Fetal insulin is elevated in ewes on a high-fat diet due to increased glucose exposure and cortisol-induced accelerated beta-cell maturation	Ford et al. (2009)
	Streptozotocin injection	Streptozotocin-induced islet beta-cell destruction causes altered maternal glucose and insulin responses and results in elevated fetal glucose, insulin, and body weight levels	Dickinson et al. (1991)
	Alloxan injection	Gestational diabetes mellitus was successfully induced in ewes by alloxan injection, but their fetuses were not significantly affected	Miodovnik et al. (1989)

## Polycystic ovary syndrome

Polycystic ovary syndrome (PCOS) is a complex endocrine disorder that is characterized by hyperandrogenemia. Hyperandrogenemia leads to abnormal follicular development, obesity, and hirsutism, which cause greater distress for women (Legro et al., 2013; Azziz et al., 2016; Lizneva et al., 2016; Rosenfield and Ehrmann, 2016; Zeng et al., 2020). However, the pathogenesis of PCOS has not yet been fully elucidated, and genetics is thought to be one of the main causes (Kahsar-Miller et al., 2001). Understanding the pathophysiology of PCOS and the methods to predict and prevent it is crucial. Because highly invasive tests are not applicable to humans, studies of PCOS need to be initiated from animal models. Although rodent models are widely used, there are differences in ovulation patterns and growth regulation between rodents and human females. In addition, there are significant differences in placental structure and fetal development between rodents and humans. Most organ development in the mouse fetus occurs after birth, which makes it less accurate to assess the effect of disease on fetal development (Carter, 2020). Because sheep and monkeys are similar to humans in terms of physiology and hormone regulation, several researchers have used these large animals to simulate human PCOS and to explore possible pathogenesis in recent years (Table 3). In a study by Tonellotto Dos Santos et al. (2018), the possible causes of the hypertrichosis symptoms caused by PCOS were investigated by injecting pregnant ewes with androgens to make them androgenic. It was found that increased 5-a-reductase type 1 (SRD5A1) activity may be a cause of hirsutism due to PCOS. PCOS is also induced in sheep by prenatal overtreatment with testosterone, and King et al. (2007) demonstrated mild hypertension in unproductive sheep after overtreatment with testosterone, which is also a symptom of PCOS. The human and sheep placentas have similar villi structures in the stem, middle and terminal vessels (Leiser et al., 1997). Kelley et al. (2019) examined the effects of testosterone treatment on the sheep placenta and demonstrated that PCOS may impair placental function through increased oxidative stress and hypoxia. In general, the higher the evolutionary stage of experimental animals, the more complex their functions and structures are, and the closer their physiological responses are to humans. Non-primate species are widely used in PCOS research (Abbott et al., 1998). In a detailed comparison of multiple animal models of PCOS in a review by Paixao et al, (2017) they concluded that the prenatal administration of androgens in non-human primates was the closest modeling approach to the real. Non-human primates treated with testosterone have the same characteristics as humans with PCOS (Abbott et al., 2016). In addition, the secretory function of the fetus is affected in individuals with PCOS. The secretion of gonadotropins is increased in fetuses of non-human primate models induced with androgens. This also provides a retrospective basis for the developmental origin of luteinizing hormone and androgen overload symptoms in adulthood (Abbott et al., 2008). Although it is costly to use large animals such as sheep and monkeys, they are suitable for androgen injection molding and the disease response is much closer to that of humans.

**TABLE 3:** Large animal models for the study of polycystic ovary syndrome.

Species	Modeling method	Result	References
Rhesus monkeys	Testosterone propionate injection	Testosterone propionate injections increased serum testosterone and androstenedione levels in maternal and prenatal androgenized fetuses	Abbott et al. (2008)
	Testosterone pellets implanting subcutaneously	Androgens stimulate gonadotropin-independent follicle growth in the primate ovary	Vendola et al. (1998)
Sheep	Testosterone propionate injection	Prenatal androgen injections reduce adipose differentiation during puberty and lead to subcutaneous adipose tissue inflammation in adulthood in sheep	Siemienowicz et al. (2021)
		Pregnant ewes showed increased hair number and diameter, mild hypertension, and impaired placental function after testosterone treatment	King et al. (2007); Tonellotto Dos Santos et al. (2018); Kelley et al. (2019)

## Endometriosis

Endometriosis is a disease caused by the growth of the endometrial glands and mesenchyme outside the uterine cavity. There are three main types of endometriosis: peritoneal endometriosis, ovarian endometriosis cysts, and deep infiltrating endometriosis (Shafrir et al., 2018; Chapron et al., 2019; Lagana et al., 2019). Li et al. (2022) were able to establish an endometriosis model in nude mice using a mixture of human immortalized endometriosis stromal cells and epithelial cells. The use of rodents to study this disease is not appropriate. In rodents, molting occurs only in the presence of a fertilized egg, whereas in humans this process is automatic. In addition, the receptors that play a dominant role in this process differ between the two (Bulun et al., 2019). However, transgenic mice are reproductively different from humans (Maenhoudt et al., 2022). Because of this, non-human primates play a large role in preclinical disease research (D’Hooghe et al., 2009). Investigators have mastered multiple methods to induce endometriosis in large animals (Table 4). Models of endometriosis formed by induction in non-human primates are highly physiologically similar to humans, and therefore many related studies have been conducted in them. Some researchers have induced endometriosis in baboons by shredding and transferring endometrial tissue to the peritoneum, ovaries, and fallopian tubes (D’Hooghe et al., 1995a). Mann et al. found that intraperitoneal implantation using menstrual endometrium rather than luteal endometrium was more successful in inducing endometriosis compared to retroperitoneal injection. They also demonstrated that continuous infusion of GnRH agonist or levonorgestrel was effective in the treatment of endometriosis in monkeys (Mann et al., 1986; Gu et al., 2020). In addition, the exploration of the molecular mechanisms of endometriosis has revealed abnormal expression of many progesterone and estrogen regulatory genes. Transient pre-disease upregulation of vascular endothelial cell growth factor-A and angiogenic factor CYR61 suggests that symptoms of endometriosis are associated with increased angiogenic capacity and imbalance of hormonal regulation (Hastings and Fazleabas, 2006). The analysis related to endometriosis in the thorax of rhesus monkeys was first reported by Assaf and Miller (2012) Immunohistochemical and other results revealed that the uterine glands and mesenchyme entered the lungs, an extremely rare case that provides a reference for the extrauterine effects of endometriosis. A comparative study of progesterone treatment versus surgical excision in rhesus monkeys showed that subcutaneous implantation is a viable treatment with a shorter recovery period compared to oral versus surgical treatment (Maginnis et al., 2008). Researchers are also using large animals to explore biological therapies for endometriosis. Adipose-Derived Stem Cells (ADSCs) have been used to study the immunomodulatory effects of ADSCs on endometriosis in mares. Although positive effects such as some infiltration of ADSCs into endometrial tissue and upregulation of matrix metalloproteinase-9 expression were observed, the upregulation of interleukin-8 may have a negative effect on the therapeutic effect (Falomo et al., 2015). Nevertheless, this study is instructive for the biological treatment of endometriosis.

**TABLE 4:** Large animal models for the study of endometriosis.

Species	Modeling method	Result	References
Cynomolgus monkeys	Shredding and transferring endometrial tissue to the peritoneum	GhRhα is therapeutically effective in monkey endometriosis, but ovulation and cessation of menstruation occur in some individuals	Werlin and Hodgen (1983); Mann et al. (1986)
	Perfusing cell suspensions of endometrial tissue into the posterior culde-sac	Intact structure is an important condition for ectopic bed of endometrial fragments, and ectopic growth is inhibited after enzymatic digestion and protease inhibitor treatment of endometrial fragments	Sillem et al. (1996)
Baboons	Intrapelvic injection of menstrual endometrium	Transplantation of menstrual endometrium is more likely to cause endometriosis than transplantation of luteal endometrium, and intraperitoneal seeding is more likely to cause endometriosis than subperitoneal injection	D’Hooghe et al. (1995b)
Marmoset monkeys	Flushing sterile medium through the fallopian tube from the uterus into the abdominal cavity	Endometriosis lesions have high local estradiol synthesis and low estrogen inactivation	Einspanier et al. (2006)
Pigs	Suturing the endometrium to the caudodorsal aspect of the bladder	Endometriosis was successfully induced in dogs, pigs and sheep by surgical transplantation of endometrium, but subsequent lesions could be observed in dogs	Varughese et al. (2018)
Dogs			
Sheep	Suturing the endometrium to caudodorsal aspect of the uterus		

## Discussion

**FIGURE 1:**
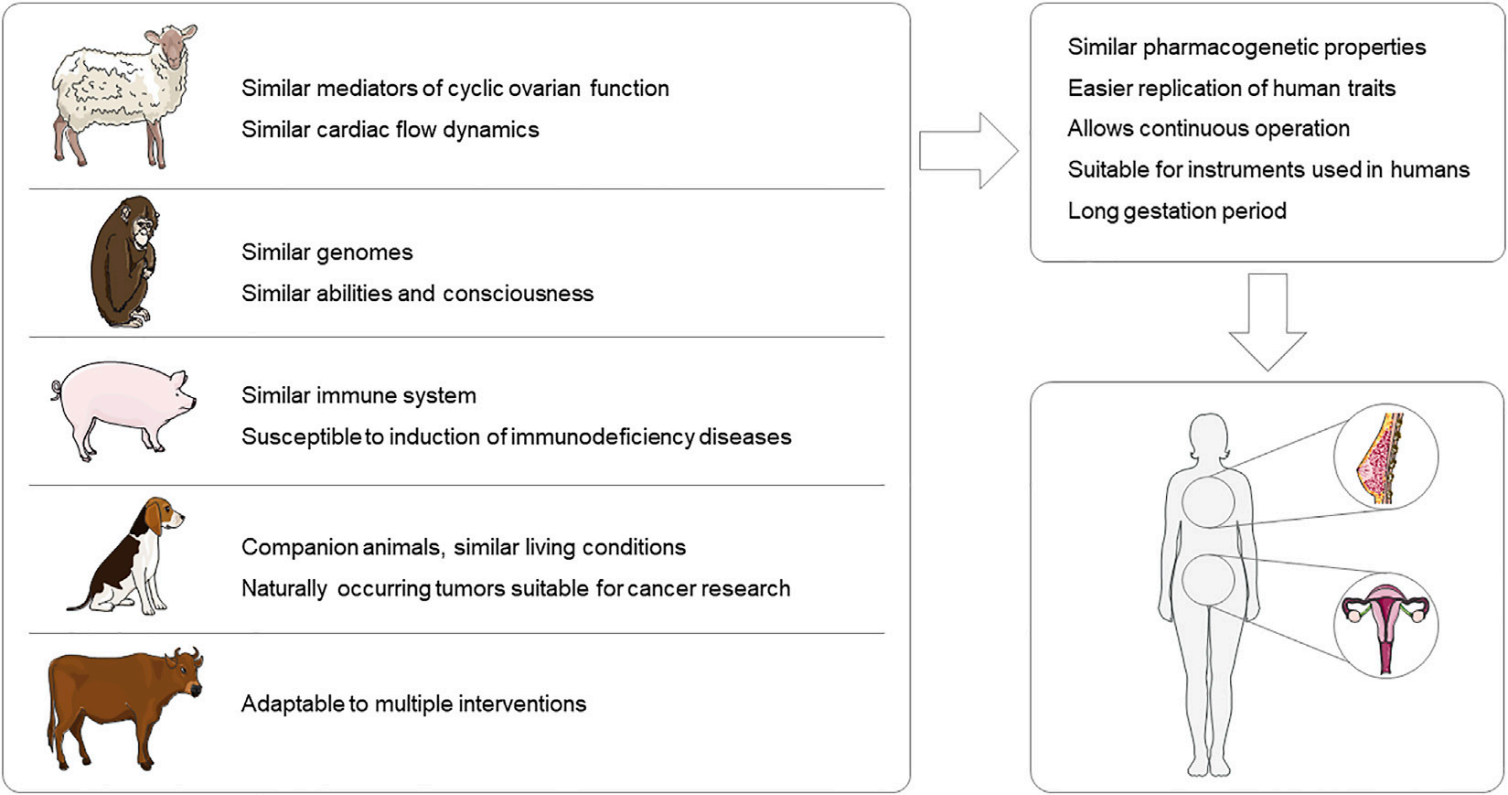
Advantages of large animal models for gynecological disease research. This figure was created using images from Servier Medical Art Commons Attribution 3.0 Unported License (http://smart.servier.com).

Large animal models are an important reliance for humans to explore disease principles, discover drug targets, and test new drugs. Small rodents such as mice and rats still account for the majority of preclinical studies in gynecological diseases. These animals are cheaper, reproduce rapidly, and the knockout technique is easier to implement compared to larger animals, allowing study results to be obtained in a short period of time. However, large animals, such as pigs, monkeys, and sheep, have reproductive organs with structures and physiological cycles that are closer to those of humans than to those of rodents. Also, the larger size of large animals and the larger volume of their organs lend themselves to a wider variety of modeling approaches and allow for real-time examination. In the study of gynecological diseases, large animal models are more suitable (Figure 1). However, there are some issues to be noted for the use of large animals. Large animals are subject to more stringent ethical review and have higher requirements for husbandry conditions. Therefore, the selection of animal models requires a comprehensive consideration by the investigator. In this review, the use of large animals in gynecological diseases is presented with three disease examples, but of course, the examples go far beyond that Trus et al. (2020) used pigs to simulate human *in utero* Zika virus infection with a pathological response highly correlated with that of humans Nahmias et al. (1971) successfully simulated genital infection with genital herpesvirus hominis type 2 virus in macaques. Researchers should be more critical in the selection of experimental animals, and the selection of appropriate animal models according to the study content is already a non-negligible item in the design of experiments.

Undeniably, with the increased use of large animal models in the study of gynecological diseases, there are many accompanying issues that deserve deeper consideration. First, although the characteristics of large animals are closer to those of humans, the issue of heterogeneity should be equally considered. There are also variations in reproductive characteristics between large animals and humans, for example, the trophoblast does not enter the uterine vasculature during pregnancy in sheep. The differences in physiological properties between large animal models and humans should be explored more deeply and these differences should be considered when studying human diseases. Finally, more attention should be paid to the welfare of large animals and to reducing the harm to experimental animals in the process of modeling and experimental validation using large animals. Though large animals are more tolerant of handling, the implementation process should follow the principles of “replace, reduce, optimize”. Since it is not feasible to conduct large-scale disease studies with humans, the use of large laboratory animals remains the better option for now. However, there are still some reproductive diseases that lack large animals as models for research, such as multiple bacterial and fungal infection models, where researchers induce infection by inoculating bacteria and fungi in the genitalia. As for large animals, more naturally occurring diseased animals have been collected for research. But there is no denying the advantages of large animals for gynecological disease research, and more modeling protocols being explored will facilitate a deeper understanding of gynecological diseases.
